# A moonshot approach toward the management of cancer patients in the COVID-19 time: what have we learned and what could the Italian network of cancer centers (Alliance Against Cancer, ACC) do after the pandemic wave?

**DOI:** 10.1186/s13046-020-01614-x

**Published:** 2020-06-11

**Authors:** Nicola Silvestris, Giovanni Apolone, Gerardo Botti, Gennaro Ciliberto, Massimo Costantini, Paolo De Paoli, Silvia Franceschi, Giuseppe Opocher, Angelo Paradiso, Paolo Pronzato, Alessandro Sgambato, Ruggero De Maria

**Affiliations:** 1 IRCCS Istituto Tumori “Giovanni Paolo II”, 70124 Bari, Italy; 2 Department of Biomedical Sciences and Human Oncology, University of Bari “Aldo Moro”, Bari, Italy; 3 Scientific Directorate, Fondazione IRCCS Istituto Tumori di Milano, Milan, Italy; 4 Scientific Directorate, Istituto Nazionale Tumori, “Fondazione G. Pascale” - IRCCS Naples, Naples, Italy; 5 Scientific Directorate, IRCCS Regina Elena National Cancer Institute, Rome, Italy; 6 Scientific Directorate, Azienda USL-IRCCS di Reggio Emilia, Reggio Emilia, Italy; 7 Alliance Against Cancer, Rome, Italy; 8 Scientific Directorate, Centro di Riferimento Oncologico di Aviano (CRO) IRCCS, Aviano, Italy; 9 Scientific Directorate, Veneto Institute of Oncology, IOV-IRCCS, Padova, Italy; 10 Scientific Directorate IRCCS Istituto Tumori “Giovanni Paolo II” of Bari, Bari, Italy; 11 Department of Oncology, IRCCS Ospedale Policlinico San Martino, Genoa, Italy; 12 Scientific Directorate IRCCS CROB Rionero in Vulture, Rionero in Vulture, Italy; 13 Fondazione Policlinico Universitario Agostino Gemelli IRCCS, Rome, Italy

**Keywords:** Cancer, COVID-19, Alliance against Cancer, Research, Therapy

## Abstract

If we focus our attention on seven main features of COVID-19 infection (*heterogeneity, fragility, lack of effective treatments and vaccines, “miraculous cures”, psychological suffering, deprivation, and globalization*), we may establish parallelism with the challenges faced in the steep road to the understanding and treatment of neoplastic diseases. How the similarities between these two conditions can help us cope with the emergency effort represented by the management of cancer patients in the COVID-19 era, today and in the future? In a manner similar to the Cancer Moonshot initiative in the United States, we can hypothesize a *multinational moonshot project towards the management of cancer patients during COVID-19 pandemic*. In particular, we believe that the main road to elaborate meaningful scientific evidence is represented by the collection of all the data on COVID-19 and cancer comorbidity that are and will become available in cancer centers, coupled with the design of large clinical studies. To address this goal, it is essential to identify the entity that can produce this scientific evidences and the potentially most successful research strategy to undertake. The largest Italian organization for cancer research, Alliance Against Cancer (*Alleanza Contro il Cancro*, ACC), is called to play a scientific leadership in addressing these challenges, which requires the coordination of oncology teams at regional, national, and international levels. To fulfill this commitment, ACC will create a liaison with health government agencies in order to develop “dynamic” indications able to fight such an unpredictable pandemic.

As of May 4th, 2020, there were 99,981 COVID-19 positive cases (16,823 hospitalized, 1479 in intensive care and 81,678 in-home isolation), and 29,079 deceased in Italy [1]. In the last week of March, the number of new laboratory-confirmed COVID-19 cases in Italy surpassed 6000 per day to decline to little more than 1000 in the first week of May. All-cause mortality in March increased by 50% at the national level and five times in the most affected province in the Lombardy region. However, it is still difficult to disentangle the direct role of COVID-19 and the indirect role of infection-related disruption on the population and health facilities [2].

In Italy, according to the estimates by AIRTUM (*Italian Association of Cancer Registries*) for 2019, there are about 1000 new cancers diagnosed and 400 cancer deaths every day with an expected prevalence of cancer patients by 2020 corresponding to about 6% of the general population [3]. To date, it is not known how many cancer patients have experienced SARS-CoV-2 infection during treatment or follow up, nor how many died from COVID-19. Moreover, we do not know how much the higher mortality rates recorded in the Italian population infected with the virus concerned the comorbidity of cancer patients. Furthermore, it is still unclear how much the social distancing measures adopted during the emergency phase, also in terms of intra- and inter-regional mobility, could have influenced the new cancer diagnoses. In March and April 2020, a 25% reduction of all cancer diagnoses (60% decrease of skin tumors) occurred in the Netherlands, a result of the combination of factors related to patients, institutions, doctors, etc. [4]. This reduction in cancer care will likely lead to an increase in cases with delayed diagnosis in more advanced stages in the future.

While waiting for accurate estimates, four months after the first cases reported in China [5] and two months after the lockdown in Italy, the question we should ask is: *what have we learned in the management of cancer patients at risk of or infected by COVID-19?* Sparse retrospective information from the hospitals that first faced this health emergency has some descriptive value but leaves many questions unanswered. According to Isaac Newton’s words: “*what we know is a drop, what we ignore is an ocean*”.

One of the few certainties is the awareness that this has been and continues to be a war on two fronts (treatment of both neoplastic diseases and COVID-19 infection), with probably some critical common issues capable of adding up not in a linear but in an exponential way. In particular, there are at least six main features of COVID-19 infection that deserve consideration as detailed below.

1. Clinical *heterogeneity*, as regards to the severity of the symptoms (from asymptomatic/pauci-symptomatic forms to extremely serious and fatal cases), clinical pictures with the involvement of different organs and systems, age of the subjects, and higher incidence in males as compared to females.
2. *Worse morbidity and mortality in the most fragile subjects*, notably elderly and/or carriers of other chronic illnesses (including malignancies and immunosuppression).
3. *Absence, to date, of effective medical remedies*, such as anti-viral treatments or vaccines capable of protecting the general population from an infection which, as in the pandemics of the past, is not only affecting healthcare but causing the worst economic and social crisis after the Second World War. Importantly, several studies are evaluating the role of sound therapeutic approaches, including tocilizumab (an IL-6 inhibitor and a potential supportive treatment for the severe respiratory symptoms associated with COVID-19), convalescent plasma therapy, and the antiviral remdesivir, which has recently received emergency approval by the US Food and Drug Administration.
4. However, regrettably, an *increasing number of treatments* proposed by unconfirmed sources and not supported by substantial evidence are being offered in COVID-19 patients outside the framework of clinical trials.
5. *Psychological suffering* related to the fear of infection and the loneliness caused by the necessary social distancing measures put in place at hospitals and homes.
6. *Social and economic deprivation* could be associated with COVID-19 onset and prognosis; in particular, the infection seems to hit the poor more often and harder than more affluent individuals.
7. The extremely rapid spread of the virus, as a result not only of its very active transmission modes but also due to *globalization*.

If we focus our attention on these seven issues (*heterogeneity, fragility, lack of effective treatments and vaccines, “miraculous cures”, psychological suffering, deprivation, and globalization*), we may establish parallelism with the challenges faced in the steep road to the understanding and treatment of neoplastic diseases. Keeping this hypothetical parallelism in mind, one could reason how the lesson from COVID-19 epidemic can be applied to cancer and vice versa, and how the similarities between the two conditions can help us cope with the emergency effort represented by the management of cancer patients in the COVID-19 era, today and in the future? In a manner similar to the Cancer Moonshot initiative in the United States [5], can we hypothesize a *multinational moonshot project toward*s *the management of cancer patients during COVID-19 pandemic*? In line with the Moonshot model, we believe that the main goal should be the *elaboration of scientific evidence* capable of supporting, on the one hand, patients and healthcare professionals by giving them certainties in their daily choices, and, on the other hand, governments and policy-makers in health policy settings.

To address this goal, it is essential to identify the entity *that* can produce this scientific evidences and the potentially most successful research strategy. The largest Italian organization for cancer research is Alliance Against Cancer (*Alleanza Contro il Cancro, ACC*), which was established in 2002 by the Italian Ministry of Health as the network of high standard institutes for patient care and research (IRCCS) in cancer [6]. Its primary aim is to promote the networking among cancer institutes pursuing mainly clinical and translational research in order to bring state of the art diagnostics and advanced therapeutics to patient care in all the Country. Every year, the Italian Cancer Centers record about 90,000 new patients, with 3500 ongoing clinical studies and about 5000 researchers involved. We believe that ACC could play a key role by promoting and coordinating the collection and processing of all those (real world) data that can help us to face the challenge represented by this pandemic that, most likely, we will have to face for a long time. Clearly, in the context of an interactive network model among networks, collaboration, and sharing of these data with the global research community will be a priority [7].

In particular, we believe that the main road to elaborate meaningful scientific evidence is represented by the collection of all the data on COVID-19 and cancer that are and will become available in cancer centers, coupled with the design of large clinical studies. In order to evaluate clinical outcomes, cancer patients admitted to various centers in pre-COVID-19 years may be used as historical controls. What data should be collected in this general research strategy?

The first operational proposal could be the collection of clinical signs and symptoms from the organs and tissues known as target of SARS-CoV-2 infection. This type of data are required to disentangle those due to the infection from those due to malignancy and improve the personalized treatment. A typical example is lung lesions visualized by diagnostic imaging. Here, the collection of data obtained with instrumental investigations can help define the radiological criteria able to differentiate SARS-CoV-2 infections from primary or metastatic lung tumors (especially pulmonary lymphangitis). The second key point would be the possibility to run clinical trials in patients who did not undergo guideline-recommended therapies during the pandemics, such as those who did not get or delayed the adjuvant treatment. Here, the involvement of general practitioners would be essential to collect data and recruit patients. The third aspect is represented by the need to analyze *pharmacological data* with the aim to learn about potential unfavorable interactions between cancer drugs and drugs used in the treatment of the COVID-19 infection. In particular, can oncological treatments in cancer patients with asymptomatic infection be considered without consequences? Can immunotherapy with checkpoint inhibitors influence the course of infection with particular regard to PD-1-related pneumonia? Can the reactivation of COVID-19 infection occur during chemotherapy? Unfortunately, pharma industries provided little or no initiatives in this context. Likewise, the EU has not been proactive for cancer patients during the pandemics and did not provide support to collaborative research, development and sharing diagnostics, clinical trials cooperation, nor to transborder assistance.

Serologic tests in cancer patients could play a crucial role since they would allow to verify the prevalence and the intensity of the immune response against SARS-CoV-2 together with the clinical impact of different treatments in different tumor types.

The systematic collection of well-annotated biological samples from COVID-19 infected cancer patients is an essential part of our proposal. It represents the best way to study the biological aspects and any long-term consequences of the infection, including a possible impact on tumor prognosis [8]. Examples of specific biological questions could be the role of the expression of the cellular entry receptor of SARS-CoV-2 (angiotensin-converting enzyme 2) in different tumor histotypes and the possible consequences of the infection. Furthermore, it will be crucial to proceed with bio-banking according to consolidated international criteria and in compliance with ethical-legal-social (ELS) requirements as well as the rights of the involved patients. Biobanking and BioMolecular Resources Research Infrastructure of Italy (BBMRI.it) has developed a shared model of informed consent and ELS tools useful for the COVID 19 research biobank, with the contribution of citizens’ representatives, patients, ethics committees, universities, research bodies, Cancer Institutes and biobanks [9]. All these data should enable us to answer crucial questions in clinical practice (i.e., what should be the correct management for a positive COVID-19 patient before or after a cycle of chemotherapy, immunotherapy, targeted therapy and/or surgery? What could be the impact of the infection on the clinical outcomes of these patients? What could be the ethical and practical implications of these data?).

IRCCS are called to play a scientific leadership in addressing these challenges that involve the coordination of oncology teams at regional, national, and international levels, to create a liaison with health government agencies and the development of “dynamic” indications fit with an unpredictable pandemic. Their mandate includes the implementation of randomized clinical trials independently of the pharmaceutical industries with the aim to avoid interferences related to various conflicts of interest involving investigators and institutions, similarly to what Italian Medicines Agency (*Agenzia Italiana del Farmaco, AIFA*) is already doing. Moreover, oncology research centers have laboratories equipped with technological equipment and instruments that can significantly contribute towards the molecular study and the control of the infection.

In conclusion, we can win this war only if we acknowledge the need for a strategy that includes what we learn from the research on both cancer and COVID-19. On these grounds, we endorse an approach similar to what we have called “liquid dynamic medicine”, i.e., the matching of dynamic changes in the tumor and dynamic changes in the therapy [10]. Indeed, the management of cancer patients during the COVID-19 pandemic will require continuous adjustments as new knowledge becomes available.

## Data Availability

Not applicable.

## Abbreviations

ACC: Alliance Against Cancer
AIFA: Agenzia Italiana del Farmaco
AIRTUM: Italian Association of Cancer Registries
BBMRI: BioMolecular Resources Research Infrastructure of Italy
ELS: ethical-legal-social
IRCCS: Istituto di Ricovero e Cura a carattere Scientifico
